# Circulating miRNAs signature on breast cancer: the MCC-Spain project

**DOI:** 10.1186/s40001-023-01471-2

**Published:** 2023-11-04

**Authors:** Inés Gómez-Acebo, Javier Llorca, Jessica Alonso-Molero, Marta Díaz-Martínez, Beatriz Pérez-Gómez, Pilar Amiano, Thalía Belmonte, Antonio J. Molina, Rosana Burgui, Gemma Castaño-Vinyals, Víctor Moreno, Ana Molina-Barceló, Rafael Marcos-Gragera, Manolis Kogevinas, Marina Pollán, Trinidad Dierssen-Sotos

**Affiliations:** 1 Department of Preventive Medicine and Public Health, University of Cantabria, Santander, Spain; 2 IDIVAL, Santander, Spain; 3 Consortium for Biomedical Research in Epidemiology and Public Health (CIBERESP), Institute of Health Carlos III, Madrid, Spain; 4 National Centre for Epidemiology, Carlos III Institute of Health, Madrid, Spain; 5 Sub Directorate for Public Health and Addictions of Gipuzkoa, Ministry of Health of the Basque Government, San Sebastian, Spain; 6 Epidemiology of Chronic and Communicable Diseases Group, Biodonostia Health Research Institute, San Sebastián, Spain; 7 IUOPA, University of Oviedo and ISPA (Health Research Institute of Asturias), Oviedo, Spain; 8 Grupo de Investigación en Interacción, Gen-Ambiente-Salud (GIIGAS), Instituto de Biomedicina (IBIOMED), Universidad de León, León, Spain; 9 Institute of Public and Occupational Health of Navarre (ISPLN), 31003 Pamplona, Spain; 10 Barcelona Institute for Global Health (ISGlobal), Barcelona, Spain; 11 Department of Medicine and Life Sciences, Universitat Pompeu Fabra (UPF), Barcelona, Spain; 12 Hospital del Mar Medical Research Institute (IMIM), Barcelona, Spain; 13 Colorectal Cancer Group, ONCOBELL Program, Instituto de Investigación Biomédica de Bellvitge (IDIBELL), Hospitalet de Llobregat, Barcelona, Spain; 14 Oncology Data Analytics Program, Catalan Institute of Oncology, Hospitalet de Llobregat, Barcelona, Spain; 15 Department of Clinical Sciences, Faculty of Medicine and health Sciences and Universitat de Barcelona Institute of Complex Systems (UBICS), University of Barcelona, Barcelona, Spain; 16 Cancer and Public Health UnitFoundation for the Promotion of Health and Biomedical Research (FISABIO-Salud Pública) in the Valencia Region, Valencia, Spain; 17 Epidemiology Unit and Girona Cancer Registry, Oncology Coordination Plan, Department of Health, Autonomous Government of Catalonia, Catalan Institute of Oncology (ICO), Girona Biomedical Research Institute (IdiBGi), Girona, Spain

**Keywords:** Breast cancer, Screening, miRNA, Diagnosis, Prognosis

## Abstract

**Purpose:**

To build models combining circulating microRNAs (miRNAs) able to identify women with breast cancer as well as different types of breast cancer, when comparing with controls without breast cancer.

**Method:**

miRNAs analysis was performed in two phases: screening phase, with a total *n* = 40 (10 controls and 30 BC cases) analyzed by Next Generation Sequencing, and validation phase, which included 131 controls and 269 cases. For this second phase, the miRNAs were selected combining the screening phase results and a revision of the literature. They were quantified using RT-PCR. Models were built using logistic regression with LASSO penalization.

**Results:**

The model for all cases included seven miRNAs (miR-423-3p, miR-139-5p, miR-324-5p, miR-1299, miR-101-3p, miR-186-5p and miR-29a-3p); which had an area under the ROC curve of 0.73. The model for cases diagnosed via screening only took in one miRNA (miR-101-3p); the area under the ROC curve was 0.63. The model for disease-free cases in the follow-up had five miRNAs (miR-101-3p, miR-186-5p, miR-423-3p, miR-142-3p and miR-1299) and the area under the ROC curve was 0.73. Finally, the model for cases with active disease in the follow-up contained six miRNAs (miR-101-3p, miR-423-3p, miR-139-5p, miR-1307-3p, miR-331-3p and miR-21-3p) and its area under the ROC curve was 0.82.

**Conclusion:**

We present four models involving eleven miRNAs to differentiate healthy controls from different types of BC cases. Our models scarcely overlap with those previously reported.

**Supplementary Information:**

The online version contains supplementary material available at 10.1186/s40001-023-01471-2.

## Background

Breast cancer is the most common type of cancer in women and a major cause of cancer death in developed countries [1]. Epidemiological research has identified several risk factors (age at menarche, parity, age at first part, age at menopause), most of them associated with estrogen production [2, 3]. Several risk factors are related to lifestyle (smoking, alcohol consumption, being overweight or obesity), although they appear to be less important than that of risk factors associated with reproductive life and estrogen production [4]. Known risk factors may explain approximately 40% of breast cancer risk.

Screening using mammograms for early diagnosis, is strongly subject to debate as observational studies suggest that its influence on breast cancer mortality is low [5]. However, identifying women at high risk of breast cancer who could benefit from different early diagnosis protocols and personalized screening is crucial. In this way, with the advent of Next Generation Sequencing (NGS) techniques, up to 313 low-penetrance genetic variants have been identified as related to breast cancer [6] and polygenic tests have been commercialized to identify women at high risk of breast cancer, although their clinical relevance is uncertain. On the other hand, the current recommendation of the St. Gallen consensus [7] is to use gene expression signatures to decide the adjuvant treatment of cancers in early stages, except in those with low clinical risk.

MicroRNAs (miRNAs) are the main class of small non-coding RNAs. Their main function is to regulate the gene expression at messenger RNA (mRNA) level [8]. In fact, it has been estimated that miRNAs regulate the expression of 30% of protein-coding genes functioning as targets of epigenetic changes or as regulators of epigenetic modifiers [8, 9]. A single miRNA can interact with quite a few mRNAs, which can have an impact on the expression of many genes at the same time [9]. More than 60% of human mRNAs contain one miRNA binding site [10]. The biological activity of individual miRNAs has been extensively studied, and the importance of their complex regulation function in many biological process has been demonstrated [8, 9]. They are involved in such vital processes for example cell proliferation, differentiation, invasion, migration, or apoptosis [9].

Any alteration in miRNAs activity (alteration in expression or in the interaction with another miRNA, for example) could be related to a variety of human diseases, including cancer [8, 9, 11]. Besides, comparing normal and tumoral tissue, miRNAs are often dysregulated in the last one [9]. In addition, it has been seen that dysregulated miRNAs could act as oncogenes (oncomiRs) or tumor suppressors [8]. However, nowadays the mechanism for the dysregulation of miRNAs in cancer is not clear but, it is possible that multiple mechanisms are at play [11]. Nonetheless, miRNAs are a good tool to diagnosis and predict prognosis in cancer patients analyzing relative miRNA expression profiles between normal and tumoral samples [9]. Although they occur in tissues, several studies have shown that tumor-specific miRNAs can be detected in the bloodstream, so in recent years interest in circulating miRNAs as non-invasive markers of disease and prognosis has been growing [12]. Specially circulating miRNAs have become potential diagnostic biomarkers in cancer given that they can easily be detected and are very robust against degradation [9]. Currently 38,589 entries from 271 organisms (1917 entries from humans) have been registered in the miRbase miRNA database [13].

Many studies carried out over the last decade have demonstrated that the dysregulation of miRNAs is present in different types of cancer, including breast cancer [10]. Breast cancer is a heterogeneous disease that involves the alteration of multiple oncogenic biological pathways and/or genetic alterations [10]. These alterations can be made by miRNAs, so researchers have performed miRNAs analysis to identify their role. For example, 64 miRNAs were identified as candidate tumor suppressor in BC cells [10]

Despite all this, the number of studies carried out on human samples is not high and much less in large cohorts. However, the identification of miRNAs as specific biomarkers would enhance early diagnosis, and personalized treatment, helping to improve breast cancer survival [10]. For this reason, the main objective in this analysis is to identify miRNAs signatures able to differentiate between controls and breast cancer cases and between controls and different types of breast cancer, using blood samples collected at recruitment in a case–control study.

## Methods

MCC-Spain is a case–control study that recruited 1738 cases of incident breast cancer in women between 2008 and 2013 as well as 1910 controls without breast cancer in 10 Spanish provinces. All cancers had been diagnosed with pathological analysis. Later, cases were follow-up until 2018 to ascertain their vital status and whether they were disease-free or not. The recruitment phase [14] and the follow-up [15, 16] have been described elsewhere. All participants signed the informed consent. The protocol of MCC-Spain was approved by the ethics committees of the participating institutions. Information about ethics and the availability of data are offered at http://www.mccspain.org. In addition, the database was registered in the Spanish Agency for Data Protection (no. 2102672171).

For the purpose of this article, breast cancer cases were classified in three categories: (A) cases diagnosed by screening (i.e., mammogram performed in asymptomatic women), as recorded at recruitment. (B) Cases diagnosed in symptomatic women who remained disease-free after the follow-up. (C) Cases diagnosed in symptomatic women who did not remain disease-free after the follow-up, but without metastases.

### Biological samples

Blood samples were obtained at recruitment from both cases and controls. Blood was centrifuged at 3000 g for 20 min at 10 °C followed by further centrifugation of the supernatant at 15000 g for 10 min at 10  C to remove cell debris. Serum was stored at – 80 °C until use.

miRNAs analysis was performed in two phases: the first phase is the screening phase and it consisted in the library preparation and Next Generation Sequencing for a small number of patients, the second phase is the validation phase and it consisted in a quantitative real-time PCR (qRT-PCR) for a larger number of patients. All experiments were conducted at QIAGEN Genomic Services.

### Screening phase

Ten control women and ten women belonging to each type of case were randomly selected for the screening phase (total *n* = 40, 10 controls, 30 cases—all of them coming from the Cantabria node and considering the three categories aforementioned) (Table 1). RNA was isolated from serum samples using the miRNeasy Serum/Plasma kit (QIAGEN) by QIAGEN Genomic Services according to manufacturer’s instructions. The library preparation was done using QIAseq miRNA Library Kit (QIAGEN), followed by quality control assessment using either Bioanalyzer2100 (Agilent) or TapeStation4200 (Agilent). A total of 200µl total RNA were converted into miRNA NGS libraries. Adapters containing Unique Molecular Index (UMIs) were ligated to the RNA, to eliminate library amplification bias. The RNA was converted to cDNA and amplified using PCR. Then, the samples were purified. The library pool was quantified using qPCR and sequenced on a NextSeq500 (Illumina). After, FASTQ files for each sample were generated. Cutadapt was used to correct PCR bias with UMI information, Bowtie2 was used for mapping the reads to Homo sapiens miRNA entries from miRbase (v22.1) and EdgeR statistical software package (Bioconductor) was used to do the differential expression analysis. Reads for each miRNA were normalized with the trimmed mean of M-values (TMM) method [17] and converted to a log2 scale to obtain delta Cq values (dCq). For this phase, the annotation of the obtained sequences was performed using the reference genome CRCh37 from the organism Homo sapiens and the annotation reference miRbase_v22.1.

**Table 1:** Number of women included in first (screening) and second (validation) phases

Group of analysis	First phase	Second phase
Controls	10	131
Cases (overall)	30	269
Cases detected by screening	10	102
Cases detected without screening, free of disease in the follow-up	10	102
Cases detected without screening, active disease in the follow-up	10	65

All the samples used in this phase have been subjected to quality controls such as: UMI collapsing (Reads need to have a unique sequence/UMI combination), high quality score and read length of > 15, be mappable to the genome CRCh37 and pass background filtering based on read numbers (removing low copy reads). If the samples do not meet these criteria, they are removed from the dataset.

### Validation phase

400 participants were randomly selected for this phase. The criterion employed was the same that in screening phase but considering that the date of blood sample collection was prior to the start date of treatment, and coming from either of the 10 Spanish provinces in MCC-Spain study. This phase included 131 controls, 102 screening-diagnosed cases, 102 disease-free cases and 65 non-disease-free cases (Table 1). The latter figure in the last group was lower than in the others because of the small total number of non-disease-free cases in the whole cohort. Fifty miRNAs were analyzed in the validation phase; they were chosen out of the results in the screening phase or for their presence in signatures already published in 2020 or 2021 [18–23]. Additional file 3: Table S1 displays the selected miRNAs and the rationale for their selection. In this phase, the serum was thawed on ice and centrifuged at 3000×*g* for 5 min in a 4 °C microcentrifuge. An aliquot of 200 µl per sample was transferred to a FluidX tube and 60 µl of Buffer RPL containing 1 µg carrier-RNA per 60 µl Buffer RPL and RNA spike-in template mixture was added to the sample and mixed for 1 min and incubated for 7 min at room temperature, followed by addition of 20 µl Buffer RPP. Total RNA was extracted from the samples using the miRNeasy Serum/Plasma Advanced kit. The purified total RNA was eluted in a final volume of 50 µl. The experiments were conducted by QIAGEN Genomic Services one more time. Later, 2 µl RNA was reverse transcribed in 10 µl reactions using the miRCURY LNA RT Kit (QIAGEN). cDNA was diluted 50 × and assayed in 10 µl PCR reactions. Each miRNA was assayed once by qPCR on the miRCURY LNA miRNA Custom PCR Panel using miRCURY LNA SYBR Green master mix (QIAGEN). Probes without RNA template from the RT step were included as negative controls and profiled like the samples. The amplification was performed in a LightCycler^®^ 480 Real-Time PCR System (Roche) in 384 well plates. The amplification curves were analyzed using the Roche LC software, both for determination of Cq (by the 2nd derivative method) and for melting curve analysis. All data was normalized to the average of custom defined assays, namely let-7d-5p and let-7i-5p, detected in all samples. As in the screening phase, reads for each miRNA were normalized with the TMM method [17] and converted to a log2 scale.

Once again, the samples used in this phase has been subjected to quality control. Data from individual reactions have been removed from the data set based on the following criteria: (i) More than one melting temperature of the amplified product; (ii) Melting temperature deviating from database values; (iii) Low amplification efficiency.

### Statistical analysis

The screening phase was analyzed by comparing dCq in controls with each type of case using the Student-t test, without any adjustment. Its results are displayed as log fold change (log FC), p-value and false discovery rate (FDR) using the Benjamini–Hochberg method (Benjamini and Hochberg 1995). LogFC positive indicate the miRNA is upregulated in cases and logFC negative that it is downregulated; the higher the absolute value of logFC, the more difference between cases and controls.

In the validation phase, we built logistic regression models with LASSO penalization [24]. LASSO penalization allows the inclusion of many regressors in the analysis while producing final parsimonious models with few regressors (miRNAs readings, in our study), as LASSO shrinks the coefficient of the less relevant to 0. To do this, we began with a model with all 50 miRNAs included in the validation phase. In the LASSO procedure, the regularization parameter *λ* was obtained via cross-validation. Then, the final models after LASSO were validated with tenfold cross-validation. Additionally to dCq, fold change and p-value, results of the validation phase are displayed as odds ratios adjusted for the remaining miRNAs in the model. The discrimination ability of each model was measured with the cross-validated mean area under the ROC curve, which is reported with its bootstrap bias corrected 95% confidence interval. In the logistic regression models, the interpretation of odds ratios would be as follows: miRNAs upregulated in cases will have odds ratios higher than 1, while miRNAs downregulated in cases will have them lower than 1. As the unit of analysis is dCq, an odds ratio of, say, 1.5 would mean that comparing two women whose dCq differ in a unity, the woman with higher dCq would have 1.5 higher odds of being a case than the woman with lower dCq. As sensitivity analysis, we reran the obtained logistic regression models with different subsets of cases: (a) breast cancers with oestrogen receptors (*n* = 212), (b) breast cancers with progesterone receptors (*n* = 187), (c) breast cancers ErbB2 positive (*n* = 50) and (d) triple negative breast cancers (*n* = 33). Of note, there is some overlapping degree between groups (a), (b) and (c). Results from this analysis are reported as area under the ROC curve with its 95% confidence interval.

All statistical analyses were carried out with the package Stata 16/SE (StataCorp, College Station, TX, US). Cross-validation was performed with the user command cvauroc[25].

### Biological functions of the selected miRNAs

The Database for Annotation, Visualization and Integrated Discovery (DAVID) version 2021 [26, 27] was used to analyse the biological functions of miRNA genes. For this purpose, sub-databases of GOTERM_BP_DIRECT, GOTERM_CC_DIRECT and GOTERM_MF_DIRECT (Gene Ontology, GO) and Kyoto Encyclopedia of Genes and Genomes (KEGG) pathway enrichment analyses were combined using DAVID online tool. In this way, we identified general miRNA functions related to regulation of gene expression (RISC complex, miRNA-mediated gene silencing and inhibition of translation, post-transcriptional gene silencing exerted by miRNAs, miRNA binding to 3′UTR regions, as well as positive and negative regulation of gene expression.

## Results

### Screening phase

The results of the quality control in this phase shown, on average, 2.4 million UMI-corrected reads were obtained for each sample and the average percentage of mappable reads was 59.3%.

**Fig. 1:**
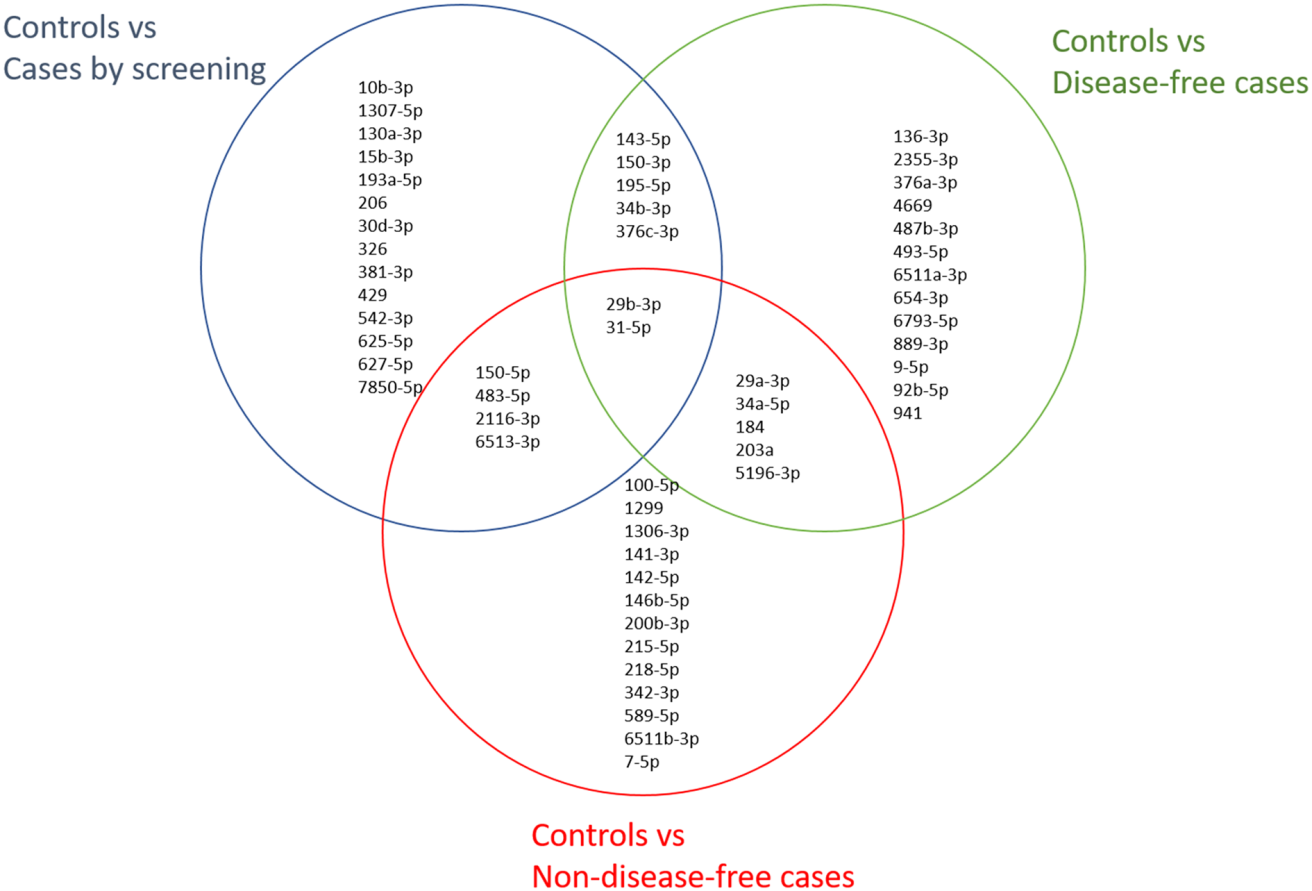
Venn diagram displaying the overlapping of miRNAs in three different comparisons: controls vs. cases diagnosed via screening, controls vs. disease-free cases and controls vs. cases with active disease in the follow-up

The 25 miRNAs showing more difference between controls and each type of case are reported in Additional file 3: Table S2 (controls vs. cases diagnosed by screening), Additional file 3: Table S3 (controls vs. disease-free cases) and Additional file 3: Table S4 (controls vs. non-disease-free cases.) There is little overlapping among these three Tables as shown in the Venn diagram in Fig. 1: Only two miRNAs (miR-29b-3p and miR-31-5p) appeared in all three, seven miRNAs came out when comparing controls vs. both cases diagnosed by screening and disease-free cases (miR-29b-3p, miR-31-5p, miR-34-3p, miR-143-5p, miR-150-3p, miR-195-5p, miR-376c-3p). Six overlapped when analyzing controls vs. cases diagnosed by screening and non-disease-free cases (miR-15b-3p, miR-206, miR-542-3p, miR-625-5p, miR-6513-3p and miR-7850-5p); and only 5 appeared when studying controls vs. both disease-free and non-disease-free cases (miR-136-3p, miR-184, miR-203a, miR-376a-3p and miR-4669).

### Validation phase

The results of the crude analysis in the validation phase appear as volcano plots in Additional file 1: Fig. S1. In each quadrant of the figure, only the miRNAs selected for the below described models are highlighted with their name.

**Fig. 2:**
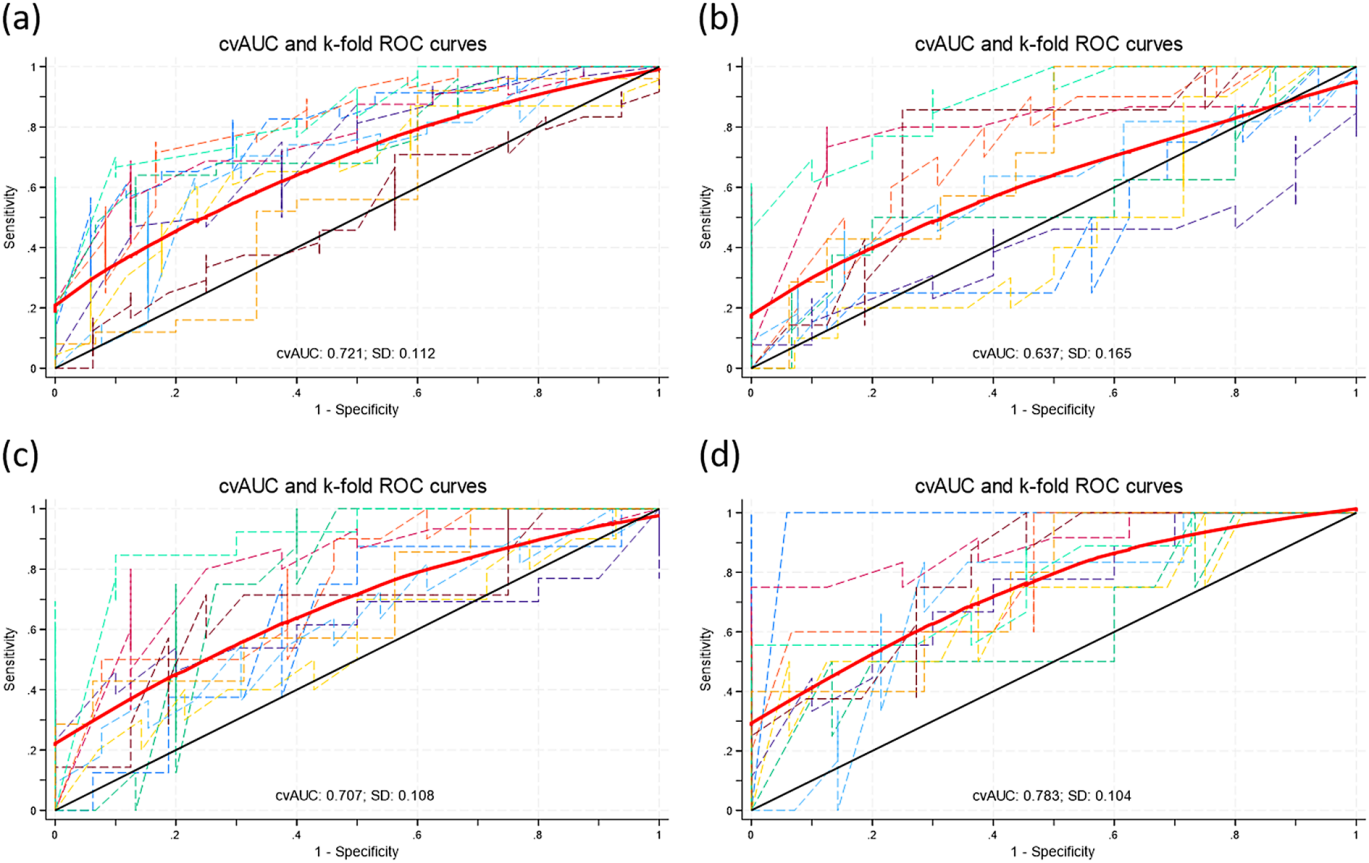
Cross-validated (solid red) and tenfold (dashed) ROC curves of the four final models. **A** Controls vs. all cases. **B** Controls vs. cases detected via screening. **C** Controls vs. disease-free cases in the follow up. **D** Controls vs. cases with active disease in the follow-up

The model comparing controls with all cases is reported in Table 2. It includes seven miRNAs: miR-423-3p, miR-139-5p, miR-324-5p and miR-1299 are upregulated (odds ratio > 1) and miR-101-3p, miR-186-5p and miR-29a-3p were downregulated in cases. The whole model has an area under the ROC curve = 0.7205 (Bootstrap bias corrected 95% CI: 0.6637–0.7773) (Fig. 2a).

**Table 2:** Signature comparing controls with cases. Final model

miRNA	TMM controls*	TMM cases*	P-value*	Fold change*	Odds ratio (95% CI)**	P**
MiR-101-3p	1.959	1.540	10^-7	0.748	0.75 (0.50, 1.12)	0.16
MiR-423-3p	− 1.283	− 1.007	2*10^-5	1.211	2.48 (1.54, 4.00)	< 0.001
MiR-139-5p	− 4.454	− 4.033	3*10^-5	1.338	1.33 (1.03, 1.71)	0.03
MiR-186-5p	− 3.510	− 3.768	0.003	0.837	0.73 (0.48, 1.11)	0.14
MiR-29a-3p	0.280	0.034	0.004	0.843	0.57 (0.39, 0.82)	0.003
MiR-324-5p	− 3.416	− 3.288	0.07	1.093	1.60 (1.08, 2.37)	0.02
miR-1299	− 6.386	− 6.146	0.01	1.181	1.30 (0.98, 1.73)	0.07

^*^Crude (unadjusted) values

^**^Adjusted for all miRNAs in the model

When comparing controls with cases detected by screening, only miR-101-3p was selected for the model, this miRNA being downregulated in cases (odds ratio = 0.52, 95% confidence interval: 0.36, 0.77) (Table 3.) The area under the ROC curve was 0.6370 (0.5209–0.6791) (Fig. 2b).

**Table 3:** Signature comparing controls with cases detected by screening. Final model

miRNA	TMM controls	TMM cases diagnosed via screening	*P*-value	Fold change	Odds ratio (95% CI)	*p*
miR-101-3p	1.959	1.635	0.0007	0.799	0.52 (0.36, 0.77)	0.001

Five miRNAs were selected for the model when comparing controls vs. disease-free cases. Two were downregulated (miR-101-3p and miR-186-5p) and three were upregulated (miR-423-3p, miR-142-3p and miR-1299) (Table 4.) The area under the ROC curve was 0.7075 (0.6180–0.7690) (Fig. 2c.)

**Table 4:** Signature comparing controls with cases disease-free in the follow-up. Final model

miRNA	TMM controls*	TMM cases*	*P*-value*	Fold change*	Odds ratio (95% CI)**	P**
miR-101-3p	1.959	1.558	8*10^-5	0.757	0.79 (0.48, 1.30)	0.35
miR-423-3p	− 1.283	− 1.037	0.003	1.186	1.97 (1.13, 3.44)	0.02
miR-142-3p	2.918	3.230	0.0002	1.242	1.55 (0.89, 2.71)	0.12
miR-186-5p	− 3.510	− 3.892	0.0006	0.768	0.60 (0.38, 0.97)	0.04
miR-1299	− 6.386	− 6.052	0.008	1.26	1.65 (1.06, 2.55)	0.03

^*^Crude (unadjusted) values

^**^Adjusted for all miRNAs in the model

The model comparing controls with cases with active disease in the follow-up included six miRNAs. miR-101-3p was strongly downregulated (odds ratio = 0.22, 95% confidence interval: 0.12, 0.43), while miR-423-3p, miR-139-5p, miR-1307-3p, miR-331-3p and miR-21-3p were upregulated in cases (Table 5.) The area under the ROC curve was 0.7835 (0.6946–0.8415) (Fig. 2d.) Results from the sensitivity analysis carried out according to the receptors present in each cancer are provided in Additional file 1: Table S5. This table should be cautiously interpreted as the sensitivity analysis could not be carried-out by cross-validation, so its results could be overfitted. Altogether, results using ErbB2-positive cases or triple negative cases tend to reach higher values in the area under the ROC curve, although confidence intervals widely overlap with those obtained with oestrogen or progesterone-positive receptors. Next, we explore the function of the 11 selected miRNAs using the DAVID bioinformatic tool. The results are displayed in Additional file 3: Table S6 (functional annotation Table for each miRNA) and Additional file 2: Figs. S2 (summary of functions involved). Six out of 11 miRNAs were involved in cancer. In particular, several functions related to angiogenesis were predicted, including positive regulation of sprouting angiogenesis, endothelial cell migration and vascular endothelial cell proliferation. Two miRNAs were associated with interleukin-1 response and with the negative regulation of beta-amyloid formation, which has been linked to cancer progression. Some biological functions related to extracellular vesicles and the extracellular space were pointed out, consistent with the fact that these miRNAs were isolated from serum. Additional file 1: Table S7 presented the miRNA sequences and their corresponding accession numbers obtained from miRBase. For a comprehensive understanding of our sample, Additional file 1: Table S8 offered a detailed description of the 269 breast cancer cases included. Furthermore, Additional file 1: Table S9 provided intricate details about the characteristics of both the cases and controls.

**Table 5:** Signature comparing controls with cases with active disease in the follow-up. Final model

miRNA	TMM controls*	TMM cases*	*P*-value*	Fold change*	Odds ratio (95% CI)**	P**
MiR-101-3p	1.959	1.364	3*10^− 8	0.662	0.22 (0.12, 0.43)	< 0.001
MiR-423-3p	− 1.283	− 0.878	2*10^− 5	1.324	1.78 (0.80, 3.95)	0.16
MiR-139-5p	− 4.454	− 3.911	0.0003	1.457	1.26 (0.80, 1.97)	0.32
MiR-1307-3p	− 4.742	− 4.176	6*10^− 5	1.480	1.49 (0.92, 2.42)	0.11
MiR-331-3p	− 3.818	− 3.413	0.0007	1.324	1.81 (0.98, 3.35)	0.06
MiR-21-3p	− 4.978	− 4.508	0.0006	1.385	1.42 (1.06, 1.91)	0.02

^*^Crude (unadjusted) values

^**^Adjusted for all miRNAs in the model

## Discussion

In this study on circulating miRNAs in breast cancer, we found models able to differentiate controls from BC cases and controls from different types of BC cases, namely cases detected by screening, cases which are disease-free in the follow-up and cases that are not disease-free in the follow-up. Although there is some degree of overlapping between the different models, it is remarkable that their calibration (i.e., their ability to discriminate between cases and controls) increases with the severity of the cancer, as shown by their areas under the ROC curve: 0.6327 to distinguish between controls and cases diagnosed by screening, 0.7345 to differentiate between controls and disease-free cases in the follow-up and 0.8216 to distinguish between controls and cases with active disease.

A total of eleven miRNAs were selected in our four models. Three miRNAs appear as downregulated (miR-101-3p, miR-186-5p and miR-29a-3p) and eight as upregulated in cases (miR-423-3p, miR-139-5p, miR-324-5p, miR-1299, miR-142-3p, miR-1307-3p, miR-331-3p and miR-21-3p).

### miRNAs downregulated in breast cancer

miR-101-3p is consistently downregulated in our four models. miR-101-3p has been described as downregulated in women with BC [28, 29]. It promotes BC cell apoptosis by targeting JAK2 (Janus kinase 2) [30] and inhibits BC growth by targeting CXCR7 (CXC chemokine receptor 7) [28] and STMN1 (Stathmin1) [29]. Harati et al. [31, 32] observe that the miR-101-3p is downregulated in metastatic breast cancer cells in comparison with less invasive cells due to the COX-2 (cyclooxygenase-2) induction. Liu et al. [33] consider that the miR-101-3p inhibits the expression of AMPK (AMP-activated protein kinase) in triple negatives breast cancer, whose dysfunction has been linked to breast cancer; while Zhao et al. [34] reflect that the overexpression of this miRNA could induce changes in the macrophages, increasing cellular proliferation and migration.

miR-186-5p appears as downregulated in our models comparing controls with all BC cases and with disease-free cases, in agreement with Giussani et al. [20]. This miRNA seem to inhibit CXCL13 (C-X-C motif chemokine ligand 13) and is associated with tumor staging and size [35]. Another way of action was raised by Hamurcu et al. [36]. They contemplate that the FOXM1 (Forkhead Box 1), which is upregulated in breast cancer cells, exerts its oncogenic effects acting over the miRNA expression. In this work, one of the miRNAs with altered expression is miR-186-5p whose upregulation is associated with the development and progression of breast cancer [36]

miR-29a-3p only appears downregulated in the model comparing controls with all BC cases. Previous results on miR-29a-3p are contradictory. While Wu et al. (2019) found a tumorigenesis role via downregulation of the histone H4K20 trimethylation, Wu et al. [37] and Li et al. [38] found it was downregulated in BC. In addition, some authors [39, 40] indicate that when the miRNA is sponged by a circRNA such as ACAP2 (circACAP2) [39] or PVT1 (Pvt1 oncogene) [40], cellular invasion, proliferation or migration increased.

### miRNAs upregulated in breast cancer

miR-423-3p is upregulated in three out of four models of ours: controls vs. all BC cases, controls vs. disease-free cases and controls vs. non-disease-free cases in the follow-up. Consistent with these results, Murria et al. [41] found that the miRNA hyperexpression is associated with estrogen or progesterone receptor positive breast cancers. In addition, the same authors [42] found that this miRNA is part of a signature, together another nine (being miR-423-3p the best differentiated), that allows discriminated hereditary and non-hereditary breast cancers. It has been experimentally observed that miR-423-3p promotes cell proliferation in BC cell lines, and its silencing leads to a decrease in cell proliferation [43]. Consistent with these results, the same authors [41] found that the miRNA hyperexpression is associated to estrogen or progesterone receptor positive breast cancers. However, it shows a lower expression in triple negative breast cancers [41]. No reference against our results was found.

Contrary to our results, miR-139-5p had previously found downregulated in BC [22, 44]. We have found no other article in agreement with our results and have no explanation for this disagreement.

Furthermore, miR-324 is upregulated when compared controls vs. all BC cases and in the comparison of controls vs. screening. In the bibliography, miR-324-5p was found upregulated in BC cases in Giusani et al. [20], Kuo et al. [45], Hong et al. [46], Lou et al. [47], and Turashvili et al. [48]. All of them have demonstrated that its upregulation is associated with worse prognosis, especially in triple negative breast cancer cancers [46–48]. Lou et al. [47] proposed a possible mechanism for this miRNA. They analyzed the GPX3 (Glutathione peroxidase 3) in BC and found that its low expression increased cell proliferation and this could be due to the release of miR-324-5p inhibition.

miR-1299 inhibits tumor cell proliferation, invasion and metastasis [49] and, so, it was found downregulated by Liu et al. [50]. This result concurs with its role in other cancers and contradicts our result which shows it as upregulated in BC. In fact, Sant et al. [51] propose that the ciRS-7 sponge the miR-1299 in triple negative breast cancer cells, leading to increase the migration and invasion cells. In the same way, Zhang et al. [52] conclude that the circ-UBR1 sponge also the miR-1299, being able to inhibit the apoptosis and facilitating the proliferation cell and metastasis.

Several authors have reported that miR-142-3p is downregulated in BC and exerts a protective role via inhibiting BC cell invasiveness [53] or targeting HMGA2 (high mobility group AT-hook 2) and inducing apoptosis [54]. These results contradict our finding of miR-142-3p as upregulated in BC. However, some authors support our results: Jusoh et al. [55] found that this miRNA was upregulated in breast cancer patients as compared to the miRNA expression of healthy subjects. In addition, Naseri et al. [56] consider that this miRNA is upregulated in many types of breast cancer resulting in the hyperproliferation of cancer cells in vitro and mammary glands in vivo.

In our results, hsa-miR-1307-3p was significantly upregulated in non-disease-free survival patients compared to controls. In the bibliography, Han et al. [57] found that the upregulation of this miRNA correlates with a poor prognosis (lower survival rate) given that this miRNA seems to stimulate cell proliferation. Shimomura et al. [58], comparing patients with breast cancer and non-breast cancer serums, conclude that a combination of five miRNA (miR-1246, miR-1307-3p, miR-4634, miR-6861-5p and miR-6875-5p) is able to detect breast cancer. Its possible mechanism has been proposed by Han et al. and Shimomura et al. who consider that the miR-1307-3p contributes to BC development and progression by targeting SMYD4 (SET and MYND domain containing 4) [57, 58]

miR-331 was overexpressed in women in metastatic BC, not only when comparing with healthy controls, but also when comparing to women with non-invasive luminal-A BC [59]. Likewise, miR-331 was overexpressed in BC with lymph node metastasis, higher TNM stage and poor prognosis [60]. These publications are consistent with our results. In addition, Pane et al. [61], using omic data integration and machine learning, anticipated that five miRNAs (mir-323a-3p, mir-323b-3p, mir-331-3p, mir-381-3p, and mir-1301-3p) could target in *EGFR (epidermal growth factor receptor)* family to develop breast cancer in the patients (among other tumors).

In our results, miR-21-3p was significantly upregulated in non-disease-free survival patients compared to controls. This is consistent with Amirfallah et al. [62], who found that its upregulation is associated with metastasis and a short disease-free survival. In addition, they found that the overexpression of this miRNA is associated with a poor prognosis. Ouyang et al. [63] also support the results. They identified 5 upregulated miRNAs (miR-155-5p, miR-21-3p, miR-181a-5p, miR-181b-5p, and miR-183-5p) when comparing the miRNAs profile expression between triple negative breast cancer and normal breast tissues. Aure et al. [64] also observed that the overexpression of three miRNAs associated with copy number gain (miR-21-3p, miR-148b-3p and miR-151a-5p) increases proliferation of breast cancer cell lines. Regarding its mechanism, some authors consider that miR-21 promotes cell proliferation and suppression of apoptosis by targeting SMAD7 (SMAD—Mothers Against decapentaplegic homolog- family member 7), PDCD4 (programmed cell death 4) and PTEN (phosphatase and tensin homolog) [65], eventually leading to increased proliferation and invasiveness of some BC [66].

As shown in both the background and the discussion sections, results on miRNA role in BC are far from homogeneous. While the role of some miRNAs (namely, miR-21, miR-101-3p, miR-186-3p, miR-331, miR-423-3p, miR-1307-3p) appears to be coherent across the literature, results on others (miR-29a-3p, miR139-5p, miR-1299 miR-142-3p) are contradictory and no clear conclusion could be reached. A similar statement could be made regarding combinations of miRNAs in models/signatures: miRNAs selected vary from model to model, making the results unreliable. For instance, only one out of five miRNAs included in the model by Shimomura et al. [58] was selected in any of our models (miR-1307-3p); Kahraman et al. (2018) [67] developed a model with seven miRNAs, but only one of them (miR-101-3p) was selected in ours; and Giussani et al. [20] obtained signatures using five miRNAs, but none was selected in our analysis. By-the-way, signatures developed by Shimomura et al. [58] Kahraman et al. [67] and Giussani et al. [20] do not share any miRNA with each other [20, 58, 67].

Explanations for this result variability would include [1] differences in statistical or lab procedures; in this regard, to select miRNAs on their crude statistical significance or using methods such as stepwise regression, which is known to inflate alpha error, could even involuntarily lead to p-hacking or cherry picking. [2] Random variability -somehow associated with the frequently small sample sizes-; and [3] true biological variability, which could be associated with diversity in the genetic background in patients studied in different countries or continents or to biological differences according to the intrinsic subtype of BC included in each study.

Our study has some limitations. Firstly, the selection of miRNAs for the validation phase was only partially based on the screening phase results, but also on previously published studies. When doing it, the authors chose miRNAs associated with BC in most recent studies (i.e., published in 2020 and 2021), but at the end the selection has some degree of subjectivity. In this way, the selection of miRNAs using their p-value in the screening phase could have led to missing some miRNAs that could have been associated with BC cases in the multivariate setting. Secondly, although beginning with a cohort of 1738 BC women, the final sample size was relatively small; this is especially true for the group of women with active disease in the follow-up, which was strongly limited out of the progressive improvement in diagnosing and treating BC. Thirdly, the discriminative power of our models is moderate as shown in areas under the ROC curve ranging 0.637 to 0.783. The study has also some strengths. Firstly, women included in the analysis were diagnosed in 10 different Spanish provinces and 23 Spanish hospitals, which guarantees some clinical variability. Secondly, our models were obtained using regression with penalization. This method (LASSO) allows for selecting parsimonious models (i.e., models with few regressors) while controlling the alpha error and avoiding the intervention of the researchers in selecting the finally included miRNAs. Moreover, LASSO is considered to outperform regression methods (e.g., stepwise) that select variables using the criticized p-value. Thirdly, we have a variety of cases (diagnosed by screening, disease-free in the follow-up and with active disease in the follow-up), which allows us to develop different models for diverse types of cases.

## Conclusion

Summarizing, we present four models involving eleven miRNAs to differentiate healthy controls from different types of BC cases. Our models scarcely overlap with those previously reported. Whether the lack of reproducibility of miRNA signatures in BC is due to methodological issues, random variability or true biological variability requires a joint analysis of data from different studies, eventually via creation of international consortia.

### Supplementary Information

**Additional file 1: Figure S1.** Volcano plots in the validation phase. **A** Controls vs. all cases. **B** Controls vs. cases detected via screening. **C** Controls vs. disease-free cases in the follow up. **D** Controls vs. cases with active disease in the follow-up

**Additional file 2: Figure S2. **Summary of functions involved in the 11 miRNAs selected in the models, according to DAVID bioinformatic tool.

**Additional file 3: Table S1**. Rationale for selecting miRNAs for the validation phase. When the rationale was based on the screening phase, the main results leading to the selection is indicated as log (fold change) and p value. When the rationale was based on previously reported results, the reference in cited. **Table S2. **Screening phase: comparison between controls and cases diagnosed by screening. Only the 25 most differentially expressed miRNAs are shown. **Table S3.** Screening phase: comparison between controls and disease-free cases. Only the 25 most differentially expressed miRNAs are shown. **Table S4.** Screening phase: comparison between controls and non-disease-free cases. Only the 25 most differentially expressed miRNAs are shown. **Table S5.** Performance of each logistic regression model according to the cancer receptor status: area under the ROC curve and 95% confidence interval. **Table S6.** Functional annotation table of the 11 miRNAs selected in the models, according to DAVID bioinformatic tool. **Table S7.** miRNA sequences and accession numbers for the validation phase obtained from miRBase. **Table S8.** Description of the 269-breast cancer included in the sample. **Table S9.** Characteristics of the breast cancer patients and population-based controls.

## Data Availability

The raw data supporting the conclusions of this article will be made available by the authors, without undue reservation.
