# Supplementary figures and images for: Circulating miRNAs signature on breast cancer: the MCC-Spain project
Source: Eur J Med Res. 2023 Nov 4;28:480. doi: 10.1186/s40001-023-01471-2 (PMC10625260; doi:10.1186/s40001-023-01471-2)

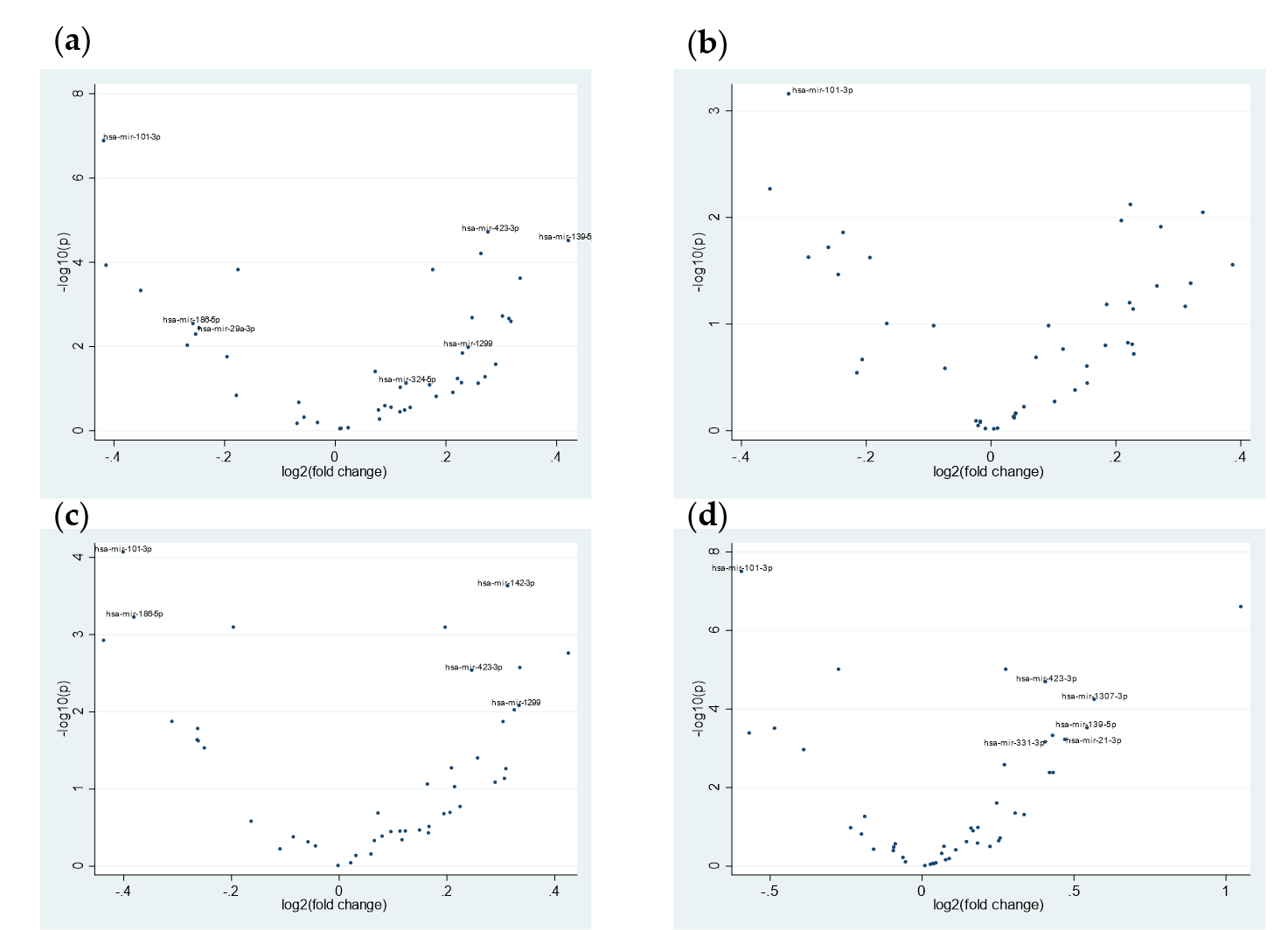

Supplement: Supplementary file 1 — Additional file 1: Figure S1. Volcano plots in the validation phase. A Controls vs. all cases. B Controls vs. cases detected via screening. C Controls vs. disease-free cases in the follow up. D Controls vs. cases with active disease in the follow-up [file 40001_2023_1471_MOESM1_ESM.tif]

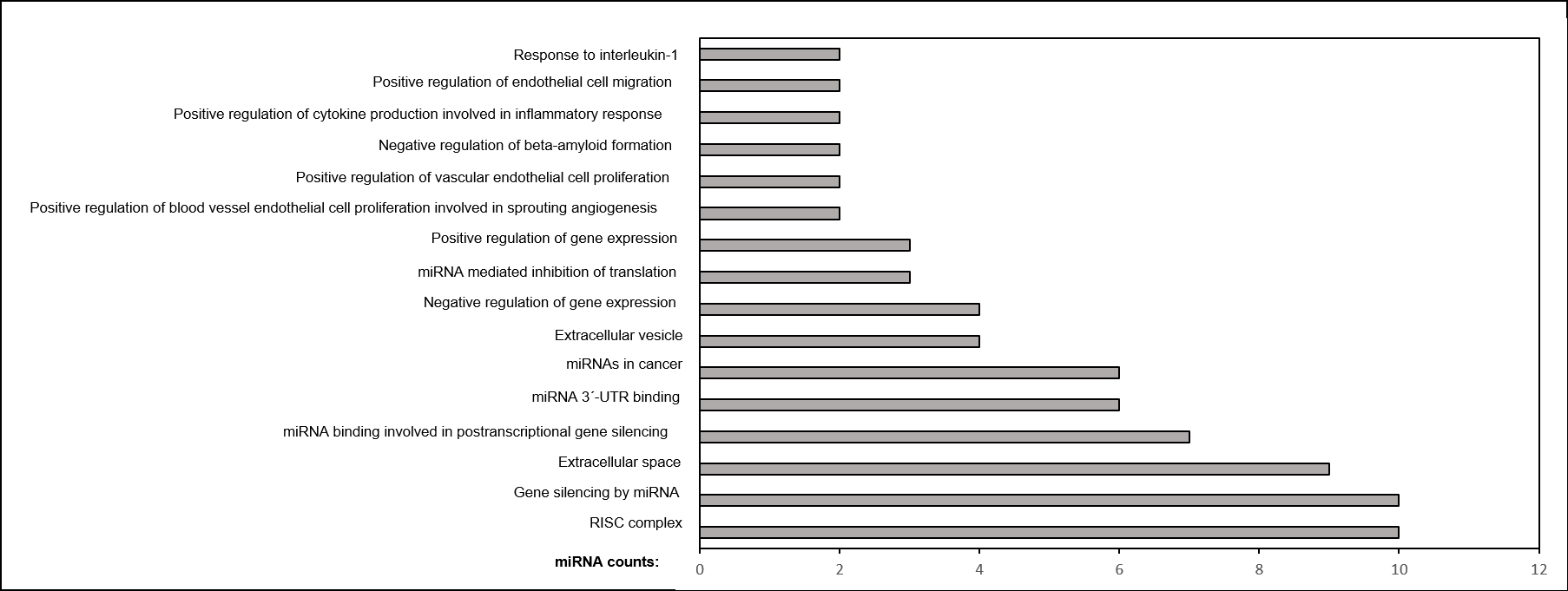

Supplement: Supplementary file 2 — Additional file 2: Figure S2. Summary of functions involved in the 11 miRNAs selected in the models, according to DAVID bioinformatic tool. [file 40001_2023_1471_MOESM2_ESM.tif]
