# Supplementary material for: Circulating miRNAs signature on breast cancer: the MCC-Spain project
Source: Eur J Med Res. 2023 Nov 4;28:480. doi: 10.1186/s40001-023-01471-2 (PMC10625260; doi:10.1186/s40001-023-01471-2)
Supplement: Supplementary file 3 — Additional file 3: Table S1. Rationale for selecting miRNAs for the validation phase. When the rationale was based on the screening phase, the main results leading to the selection is indicated as log (fold change) and p value. When the rationale was based on previously reported results, the reference in cited. Table S2. Screening phase: comparison between controls and cases diagnosed by screening. Only the 25 most differentially expressed miRNAs are shown. Table S3. Screening phase: comparison between controls and disease-free cases. Only the 25 most differentially expressed miRNAs are shown. Table S4. Screening phase: comparison between controls and non-disease-free cases. Only the 25 most differentially expressed miRNAs are shown. Table S5. Performance of each logistic regression model according to the cancer receptor status: area under the ROC curve and 95% confidence interval. Table S6. Functional annotation table of the 11 miRNAs selected in the models, according to DAVID bioinformatic tool. Table S7. miRNA sequences and accession numbers for the validation phase obtained from miRBase. Table S8. Description of the 269-breast cancer included in the sample. Table S9. Characteristics of the breast cancer patients and population-based controls. [file 40001_2023_1471_MOESM3_ESM.docx]

**Additional file**

**Additional file table 1**. Rationale for selecting miRNAs for the validation phase. When the rationale was based on the screening phase, the main results leading to the selection is indicated as log (fold change) and p value. When the rationale was based on previously reported results, the reference in cited.

|  | **Result in screening phase, controls vs. each type of case** | | |  |
| --- | --- | --- | --- | --- |
| **miRNA** | **Cases diagnosed by screening** | **Cases disease-free in the follow-up** | **Cases non-disease-free in the follow-up** | **Reference** |
| **miR-1-3p** |  |  | Log FC=-0.99  P=0.02 | Galvão-Lima et al (2021)[19] |
| **miR-7-5p** |  |  | Log FC=-0.58  P=0.02 | Liu et al (2020)  Giussani et al (2021) [20,23] |
| **miR-18a-5p** |  |  |  | Giussani et al (2021)[20] |
| **miR-21-3p** |  |  |  | Galvão-Lima et al (2021) Giussani et al (2021) [19,20] |
| **miR-21-5p** |  |  |  | Galvão-Lima et al (2021) Giussani et al (2021) [19,20] |
| **miR-26a-3p** |  |  |  | Galvão-Lima et al (2021)  Escuin et al (2021) [18,19] |
| **miR-29a-3p** |  | Log FC=-1.22  P=0.02 | Log FC=-1.60  P=0.002 |  |
| **miR-34a-5p** |  | Log FC=1.52  P=0.03 | Log FC=1.13  P=0.04 |  |
| **miR-101-3p** |  |  |  | Escuin et al (2021)[18] |
| **miR-133a-3p** |  |  |  | Escuin et al (2021) [18] |
| **miR-134-5p** |  |  |  | Giussani et al (2021) [20] |
| **miR-139-5p** |  |  |  | Liu et al (2020) [23] |
| **miR-141-3p** |  |  | Log FC=-1.76  P=0.0001 | Liu et al (2020)[23] |
| **miR-142-3p** |  |  |  | Giussani et al (2021)[20] |
| **miR-144-3p** |  |  |  | Escuin et al (2021)[18] |
| **miR-146a-5p** |  |  |  | Li et al (2020) [22] |
| **miR-148a-3p** |  |  |  | Giussani et al (2021)  Li et al (2020) [20,22] |
| **miR-150-3p*** | Log FC=-1.36  P=0.0005 | Log FC=-1.05  P=0.008 |  |  |
| **miR-181c-5p*** |  |  |  | Giussani et al (2021) [20] |
| **miR-184** |  | Log FC=-1.77  P=0.003 | Log FC=-1.82  P=0.002 |  |
| **miR-186-5p** |  |  |  | Giussani et al (2021)[20] |
| **miR-203a-3p** |  | Log FC=-1.96  P=0.007 | Log FC=-2.07  P=0.005 | Galvão-Lima et al (2021) [19] |
| **miR-206** |  |  | Log FC=-1.64  P=0.004 | Jang et al (2021) [21] |
| **miR-215-5p** |  |  | Log FC=-1.19  P=0.008 |  |
| **miR-218-5p** |  |  |  | Giussani et al (2021) [20] |
| **miR-301b-3p** |  |  |  | Giussani et al (2021) [20] |
| **miR-324-3p** |  |  |  | Giussani et al (2021) [20] |
| **miR-324-5p** |  |  |  | Giussani et al (2021) [20] |
| **miR-326** | Log FC=-1.08  P=0.005 |  |  | Escuin et al (2021) [18] |
| **miR-328-3p** |  |  |  | Escuin et al (2021) [18] |
| **miR-331-3p** |  |  |  | Galvão-Lima et al (2021) [19]  Escuin et al (2021) [18] |
| **miR-339-5p** |  |  |  | Escuin et al (2021) [18] |
| **miR-369-3p** |  |  |  | Escuin et al (2021) [18] |
| **miR-370-5p** |  |  |  | Liu et al (2020) [23]  Giussani et al (2021) |
| **miR-376a-3p** |  | Log FC=-2.00  P=0.004 |  | Liu et al (2020) [23] |
| **miR-423-3p** |  |  |  | Escuin et al (2021) [18] |
| **miR-423-5p** |  |  |  | Giussani et al (2021) [20] |
| **miR-493-3p** |  |  |  | Escuin et al (2021) [18] |
| **miR-502-3p** |  |  |  | Giussani et al (2021) [20] |
| **miR-548c-5p** |  |  |  | Block et al (2018) [67] |
| **miR-625-5p** | Log FC=-1.20  P=0.002 |  |  | Giussani et al (2021) [20] |
| **miR-664a-5p** |  |  |  | Escuin et al (2021) [18] |
| **miR-885-3p** |  |  | Log FC=1.85, p=0.001 |  |
| **miR-1307-3p** |  |  |  | Escuin et al (2021) [18] |
| **miR-1299** |  |  | Log FC=1.78  P=0.03 |  |
| **hsa-let-7d-5p** |  |  |  | Galvão-Lima et al (2021) [19] |
| **Hsa-let-7i-5p** |  |  |  | Banerjee et al (2020) [68] |
| **Hsa-190b-5p** |  |  |  | Dai et al (2019) [69] |

Log FC: log of fold change. Negative figures in log FC indicate the miRNA is under-regulated in cases. Positive figures in log FC indicate the miRNA is up-regulated in cases

**Additional file Table 2.** Screening phase: comparison between controls and cases diagnosed by screening. Only the 25 most differentially expressed miRNAs are shown

| **Name** | **LogFC** | **Cases diagnosed by screening (TMM)** | **Controls (TMM)** | **P value** | **FDR** |
| --- | --- | --- | --- | --- | --- |
| **miR-6513-3p** | -1.69 | 2 | 10 | 0.0005 | 0.1017 |
| **miR-150-3p** | -1.36 | 18 | 48 | 0.0005 | 0.1017 |
| **miR-31-5p** | -2.77 | 4 | 29 | 0.0011 | 0.1548 |
| **miR-625-5p** | -1.20 | 7 | 16 | 0.0021 | 0.2103 |
| **miR-10b-3p** | 1.60 | 8 | 2 | 0.0028 | 0.2256 |
| **miR-15b-3p** | -0.86 | 39 | 71 | 0.0041 | 0.2768 |
| **miR-326** | -1.08 | 46 | 97 | 0.0054 | 0.3131 |
| **miR-130a-3p** | -0.66 | 87 | 137 | 0.0074 | 0.3774 |
| **miR-542-3p** | 1.23 | 14 | 6 | 0.0091 | 0.3804 |
| **miR-195-5p** | 0.91 | 39 | 21 | 0.0099 | 0.3804 |
| **miR-34b-3p** | -2.51 | 0 | 3 | 0.0103 | 0.3804 |
| **miR-143-5p** | 1.48 | 6 | 2 | 0.0114 | 0.3850 |
| **miR-376c-3p** | -1.58 | 5 | 14 | 0.0143 | 0.4236 |
| **miR-483-5p** | 1.21 | 623 | 269 | 0.0146 | 0.4236 |
| **miR-2116-3p** | 1.66 | 8 | 3 | 0.0183 | 0.4376 |
| **miR-206** | 1.39 | 90 | 34 | 0.0202 | 0.4376 |
| **miR-7850-5p** | 2.24 | 3 | 0 | 0.0204 | 0.4376 |
| **miR-429** | 1.86 | 9 | 2 | 0.0204 | 0.4376 |
| **miR-29b-3p** | -1.16 | 196 | 438 | 0.0231 | 0.4376 |
| **miR-1307-5p** | -0.76 | 32 | 54 | 0.0233 | 0.4376 |
| **miR-193a-5p** | 0.89 | 435 | 236 | 0.0234 | 0.4376 |
| **miR-150-5p** | -0.76 | 4178 | 7075 | 0.0237 | 0.4376 |
| **miR-381-3p** | -1.14 | 21 | 44 | 0.0328 | 0.5624 |
| **miR-30d-3p** | -1.39 | 1 | 3 | 0.0337 | 0.5624 |
| **miR-627-5p** | -1.25 | 2 | 6 | 0.0347 | 0.5624 |

FC: Fold Change

TMM: Trimmed mean of M-values

FDR: False Discovery Rate (Benjamini-Hochberg)

**Additional file Table 3.** Screening phase: comparison between controls and disease-free cases. Only the 25 most differentially expressed miRNAs are shown

| **Name** | **LogFC** | **Disease-free cases (TMM)** | **Controls (TMM)** | **P value** | **FDR** |
| --- | --- | --- | --- | --- | --- |
| **miR-136-3p** | -2.44 | 1 | 9 | 0.0005 | 0.1999 |
| **miR-92b-5p** | 0.98 | 46 | 24 | 0.0015 | 0.2164 |
| **miR-143-5p** | 1.93 | 10 | 2 | 0.0019 | 0.2164 |
| **miR-5196-3p** | -2.69 | 0 | 4 | 0.0021 | 0.2164 |
| **miR-184** | -1.77 | 17 | 61 | 0.0032 | 0.2214 |
| **miR-376a-3p** | -2.00 | 3 | 12 | 0.0036 | 0.2214 |
| **miR-376c-3p** | -1.73 | 3 | 13 | 0.0038 | 0.2214 |
| **miR-9-5p** | -1.89 | 1 | 5 | 0.0047 | 0.2422 |
| **miR-203a** | -1.96 | 41 | 160 | 0.0074 | 0.3326 |
| **miR-150-3p** | -1.05 | 23 | 47 | 0.0081 | 0.3326 |
| **miR-34b-3p** | -2.23 | 0 | 4 | 0.0093 | 0.3450 |
| **miR-941** | 0.61 | 211 | 140 | 0.0149 | 0.4952 |
| **miR-29b-3p** | -1.22 | 176 | 409 | 0.0162 | 0.4952 |
| **miR-493-5p** | -1.19 | 4 | 10 | 0.0169 | 0.4952 |
| **miR-34a-5p** | 1.52 | 55 | 19 | 0.0251 | 0.6833 |
| **miR-29a-3p** | -1.11 | 1658 | 3566 | 0.0287 | 0.7339 |
| **miR-6511a-3p** | 1.22 | 13 | 5 | 0.0336 | 0.8081 |
| **miR-2355-3p** | 0.80 | 6 | 2 | 0.0364 | 0.8272 |
| **miR-31-5p** | -1.92 | 7 | 28 | 0.0415 | 0.8928 |
| **miR-654-3p** | -1.00 | 74 | 148 | 0.0466 | 0.9520 |
| **miR-889-3p** | -1.28 | 5 | 12 | 0.0498 | 0.9520 |
| **miR-6793-5p** | 1.26 | 4 | 1 | 0.0512 | 0.9520 |
| **miR-4669** | 1.42 | 7 | 2 | 0.0641 | 1.0000 |
| **miR-487b-3p** | -1.13 | 13 | 28 | 0.0666 | 1.0000 |
| **miR-195-5p** | 0.61 | 32 | 21 | 0.0671 | 1.0000 |

FC: Fold Change

TMM: Trimmed mean of M-values

FDR: False Discovery Rate (Benjamini-Hochberg)

**Additional file Table 4.** Screening phase: comparison between controls and non-disease-free cases. Only the 25 most differentially expressed miRNAs are shown

| **Name** | **LogFC** | **Non-disease-free cases (TMM)** | **Controls (TMM)** | **P value** | **FDR** |
| --- | --- | --- | --- | --- | --- |
| **miR-215-5p** | -1.76 | 18 | 60 | 0.0003 | 0.1178 |
| **miR-542-3p** | -1.50 | 5 | 15 | 0.0006 | 0.1215 |
| **miR-15b-3p** | 0.80 | 70 | 40 | 0.0037 | 0.3125 |
| **miR-206** | -1.64 | 29 | 90 | 0.0038 | 0.3125 |
| **miR-7850-5p** | -1.98 | 0 | 3 | 0.0039 | 0.3125 |
| **miR-148a-5p** | -1.47 | 1 | 5 | 0.0048 | 0.3217 |
| **miR-375** | -1.12 | 236 | 511 | 0.0059 | 0.3362 |
| **miR-218-5p** | -2.12 | 1 | 6 | 0.0077 | 0.3362 |
| **miR-4669** | 2.18 | 7 | 1 | 0.0081 | 0.3362 |
| **miR-625-5p** | 0.96 | 14 | 7 | 0.0084 | 0.3362 |
| **miR-5698** | 1.81 | 3 | 0 | 0.0105 | 0.3695 |
| **miR-184** | -1.06 | 17 | 37 | 0.0116 | 0.3695 |
| **miR-625-3p** | 0.74 | 472 | 282 | 0.0119 | 0.3695 |
| **miR-194-5p** | -0.67 | 261 | 418 | 0.0131 | 0.3749 |
| **miR-6820-3p** | -1.25 | 1 | 3 | 0.0171 | 0.4253 |
| **miR-556-3p** | -1.94 | 0 | 3 | 0.0177 | 0.4253 |
| **miR-192-5p** | -0.73 | 412 | 688 | 0.0180 | 0.4253 |
| **miR-200a-3p** | -1.20 | 10 | 25 | 0.0199 | 0.4264 |
| **miR-6513-3p** | 1.15 | 6 | 2 | 0.0209 | 0.4264 |
| **miR-376a-3p** | -1.25 | 3 | 8 | 0.0212 | 0.4264 |
| **miR-548ay-5p** | -1.03 | 1 | 4 | 0.0252 | 0.4683 |
| **miR-1** | -0.81 | 24 | 43 | 0.0256 | 0.4683 |
| **miR-629-3p** | -1.36 | 1 | 4 | 0.0270 | 0.4715 |
| **miR-203a** | -1.09 | 41 | 88 | 0.0292 | 0.4891 |
| **miR-136-3p** | -1.22 | 1 | 4 | 0.0323 | 0.5097 |

FC: Fold Change

TMM: Trimmed mean of M-values

FDR: False Discovery Rate (Benjamini-Hochberg)

**Additional file Table 5.** Performance of each logistic regression model according to the cancer receptor status: area under the ROC curve and 95% confidence interval

| Model | All cases* | Oestrogen receptor + | Progesterone receptor + | ErbB2 + | Triple negative |
| --- | --- | --- | --- | --- | --- |
| Cases vs. controls | 0.721 (0.664, 0.777) | 0.721 (0.662, 0.776) | 0.720 (0.663, 0.777) | 0.783 (0.712, 0.854) | 0.791 (0.714, 0.868) |
| Cases detected by screening vs. controls | 0.637 (0.521, 0.679) | 0.630 (0.551, 0.708) | 0.629 (0.547, 0.712) | 0.659 (0.518, 0.801) | 0.599 (0.387, 0.812) |
| Cases disease-free the follow-up vs. controls | 0.708 (0.618, 0.769) | 0.724 (0.655, 0.793) | 0.730 (0.659, 0.801) | 0.801 (0.714, 0.896) | 0.841 (0.737, 0.946) |
| Cases with active disease in the follow-up vs. controls | 0.784 (0.695, 0.783) | 0.813 (0.736, 0.890) | 0.825 (0.750, 0.900) | 0.868 (0.745, 0.991) | 0.889 (0.802, 0.977) |

*Results analysing all cases are included as reference; they represent mean of cross-validation estimates (see the text). Results using subsets of cases were obtained by rerunning logistic regression models (i.e., without cross-validation), as some folds in the cross-validation procedure showed no outcome variability. This puts a note of concern as these estimates could be overfitted.

**Additional file Table 6**. Functional annotation table of the 11 miRNAs selected in the models, according to DAVID bioinformatic tool.

| **miRNA** | **Biological functions** |
| --- | --- |
| miR-101-3p | Positive regulation of gene expression, negative regulation of gene expression, negative regulation of protein ubiquitination, negative regulation of interleukin-1 beta production, negative regulation of interleukin-6 production, negative regulation of tumor necrosis factor production, gene silencing by miRNA, miRNA mediated inhibition of translation, negative regulation of amyloid precursor protein biosynthetic process, negative regulation of sequence-specific DNA binding transcription factor activity, negative regulation of blood vessel endothelial cell migration, protein stabilization, negative regulation of cell adhesion molecule production, negative regulation of necroptotic process, response to interleukin-1, positive regulation of cell migration involved in sprouting angiogenesis, positive regulation of blood vessel endothelial cell proliferation involved in sprouting angiogenesis, positive regulation of endothelial cell apoptotic process, extracellular space, RISC complex, extracellular vesicle, mRNA 3'-UTR binding, mRNA binding involved in posttranscriptional gene silencing, microRNAs in cancer. |
| miR-1307-3p | Gene silencing by miRNA, extracellular space, RISC complex, extracellular vesicle. |
| miR-139-5p | Gene silencing by miRNA, extracellular space, RISC complex, mRNA 3'-UTR binding, mRNA binding involved in posttranscriptional gene silencing. |
| miR-142-3p | Response to lipopolysaccharide, negative regulation of interleukin-1 alpha production, response to tumor necrosis factor, gene silencing by miRNA, negative regulation of inflammatory response, regulation of synaptic transmission, glutamatergic, response to interleukin-1, negative regulation of bicellular tight junction assembly, positive regulation of microglial cell activation, positive regulation of membrane permeability, extracellular space, RISC complex, mRNA 3'-UTR binding, mRNA binding involved in posttranscriptional gene silencing. |
| miR-186-5p | Negative regulation of gene expression, negative regulation of transporter activity, gene silencing by miRNA, positive regulation of intrinsic apoptotic signaling pathway by p53 class mediator, negative regulation of beta-amyloid formation, negative regulation of drug transmembrane export, extracellular space, RISC complex, mRNA 3'-UTR binding, mRNA binding involved in posttranscriptional gene silencing. |
| miR-21-3p | Blood vessel development, positive regulation of cytokine production, positive regulation of protein phosphorylation, negative regulation of endothelial cell proliferation, negative regulation of cytokine-mediated signaling pathway, transforming growth factor beta receptor signaling pathway, embryo implantation, positive regulation of cell proliferation, negative regulation of cell proliferation, regulation of cell shape, positive regulation of endothelial cell migration, negative regulation of endothelial cell migration, positive regulation of cardiac muscle hypertrophy, negative regulation of cardiac muscle hypertrophy, positive regulation of gene expression, negative regulation of gene expression, negative regulation of cardiac muscle cell apoptotic process, positive regulation of epithelial to mesenchymal transition, positive regulation of keratinocyte proliferation, positive regulation of phosphatidylinositol 3-kinase signaling, negative regulation of angiogenesis, regulation of cell migration, positive regulation of cell migration, negative regulation of cell migration, BMP signaling pathway, positive regulation of transforming growth factor beta receptor signaling pathway, positive regulation of cell projection organization, negative regulation of NF-kappaB transcription factor activity, activation of protein kinase B activity, negative regulation of type I interferon production, negative regulation of interleukin-12 production, negative regulation of interleukin-21 production, collagen metabolic process, negative regulation of GTPase activity, negative regulation of smooth muscle cell apoptotic process, gene silencing by miRNA, miRNA mediated inhibition of translation, phenotypic switching, regulation of epidermal growth factor receptor signaling pathway, positive regulation of activated T cell proliferation, positive regulation of apoptotic process, negative regulation of apoptotic process, negative regulation of MAPK cascade, negative regulation of regulatory T cell differentiation, positive regulation of fat cell differentiation, positive regulation of endothelial cell differentiation, positive regulation of osteoblast differentiation, positive regulation of angiogenesis, negative regulation of innate immune response, negative regulation of mitotic cell cycle, positive regulation of inflammatory response, negative regulation of stress fiber assembly, regulation of protein kinase B signaling, positive regulation of protein kinase B signaling, negative regulation of type I interferon-mediated signaling pathway, epithelial to mesenchymal transition involved in cardiac fibroblast development, negative regulation of cartilage development, endothelial tube morphogenesis, negative regulation of ERK1 and ERK2 cascade, positive regulation of ERK1 and ERK2 cascade, cellular response to lipopolysaccharide, cellular response to gamma radiation, cellular response to virus, positive regulation of cytokine production involved in inflammatory response, positive regulation of cellular response to hypoxia, negative regulation of arginine catabolic process, positive regulation of vascular endothelial growth factor signaling pathway, negative regulation of NIK/NF-kappa B signaling, negative regulation of hydrogen peroxide-mediated programmed cell death, negative regulation of superoxide dismutase activity, negative regulation of intrinsic apoptotic signaling pathway by p53 class mediator, regulation of calcium ion transmembrane transport via high voltage-gated calcium channel, negative regulation of chondrocyte proliferation, negative regulation of reactive oxygen species biosynthetic process, positive regulation of blood vessel endothelial cell proliferation involved in sprouting angiogenesis, positive regulation of potassium ion export across plasma membrane, cellular response to resveratrol, positive regulation of metalloendopeptidase activity, positive regulation of vascular smooth muscle contraction, positive regulation of vascular smooth muscle cell proliferation, positive regulation of replicative senescence, negative regulation of vascular associated smooth muscle cell migration, positive regulation of vascular associated smooth muscle cell migration, positive regulation of myofibroblast differentiation, negative regulation of hydrogen sulfide biosynthetic process, negative regulation of aortic smooth muscle cell differentiation, positive regulation of leukocyte adhesion to vascular endothelial cell, positive regulation of vascular smooth muscle cell differentiation, negative regulation of vascular associated smooth muscle cell apoptotic process, positive regulation of vascular endothelial cell proliferation, negative regulation of endothelial tube morphogenesis, retinal cell apoptotic process, positive regulation of T-helper 17 type immune response, positive regulation of T-helper 17 cell differentiation, regulation of reactive oxygen species metabolic process, negative regulation of interleukin-1-mediated signaling pathway, extracellular space, cytoplasm, RISC complex, extracellular exosome, extracellular vesicle, mRNA 3'-UTR binding, mRNA binding involved in posttranscriptional gene silencing, proteoglycans in cancer, microRNAs in cancer. |
| miR-29a-3p | Negative regulation of cell proliferation, positive regulation of endothelial cell migration, positive regulation of gene expression, negative regulation of gene expression, negative regulation of angiogenesis, positive regulation of cell migration, negative regulation of cell migration, negative regulation of collagen biosynthetic process, gene silencing by miRNA, miRNA mediated inhibition of translation, positive regulation of apoptotic process, regulation of DNA methylation, positive regulation of epidermal growth factor receptor signaling pathway, positive regulation of angiogenesis, positive regulation of protein kinase B signaling, negative regulation of protein kinase B signaling, negative regulation of circulating fibrinogen levels, positive regulation of wound healing, positive regulation of G1/S transition of mitotic cell cycle, positive regulation of mitochondrial membrane permeability involved in apoptotic process, negative regulation of beta-amyloid formation, negative regulation of amyloid precursor protein catabolic process, positive regulation of vascular endothelial cell proliferation, negative regulation of G1/S transition of mitotic cell cycle, extracellular space, cytoplasm, mitochondrion, RISC complex, extracellular exosome, extracellular vesicle, mRNA 3'-UTR binding, mRNA binding involved in posttranscriptional gene silencing, microRNAs in cancer, chemical carcinogenesis - receptor activation. |
| miR-324-5p | Sensory perception of sound, gene silencing by miRNA, long-term synaptic potentiation, positive regulation of cytokine production involved in inflammatory response, cellular response to leukemia inhibitory factor, RISC complex, microRNAs in cancer. |
| miR-331-3p | Gene silencing by miRNA, extracellular space, RISC complex, microRNAs in cancer. |
| miR-423-3p | Gene silencing by miRNA, extracellular space, RISC complex, extracellular exosome, mRNA binding involved in posttranscriptional gene silencing, microRNAs in cancer. |

**Additional file Table 7**. miRNA sequences and accession numbers for the validation phase obtained from miRBase.

| **miRNA** | **miRNA sequence** | **Mature miRNA miRBase accession number** |
| --- | --- | --- |
| hsa-miR-21-3p | CAACACCAGUCGAUGGGCUGU | MIMAT0004494 |
| hsa-miR-29a-3p | UAGCACCAUCUGAAAUCGGUUA | MIMAT0000086 |
| hsa-miR-101-3p | UACAGUACUGUGAUAACUGAA | MIMAT0000099 |
| hsa-miR-139-5p | UCUACAGUGCACGUGUCUCCAGU | MIMAT0000250 |
| hsa-miR-142-3p | UGUAGUGUUUCCUACUUUAUGGA | MIMAT0000434 |
| hsa-miR-186-5p | CAAAGAAUUCUCCUUUUGGGCU | MIMAT0000456 |
| hsa-miR-331-3p | GCCCCUGGGCCUAUCCUAGAA | MIMAT0000760 |
| hsa-miR-324-5p | CGCAUCCCCUAGGGCAUUGGUG | MIMAT0000761 |
| hsa-miR-423-3p | AGCUCGGUCUGAGGCCCCUCAGU | MIMAT0001340 |
| hsa-miR-1299 | UUCUGGAAUUCUGUGUGAGGGA | MIMAT0005887 |
| hsa-miR-1307-3p | ACUCGGCGUGGCGUCGGUCGUG | MIMAT0005951 |

**Additional file Table 8**. Description of the 269-breast cancer included in the sample.

|  | Cases detected via screening | Disease-free cases in the follow-up | Cases with active disease in the follow-up | P-value |
| --- | --- | --- | --- | --- |
|  | 102 (37.9%) | 102 (37.9%) | 65 (24.2%) |  |
| Age mean (SD) | 57.5 ± 11.7 | 54.5 ± 13.8 | 55.2 ± 14.4 | 0.246 |
| Histology |  |  |  |  |
| Ductal | 71 (72.4%) | 85 (83.3%) | 53 (81.5%) | 0.046 |
| Lobular | 4 (4.1%) | 7 (6.9%) | 6 (9.2%) |  |
| Papilar | 1 (1.0%) | 2 (2.0%) | 1 (1.5%) |  |
| Others | 22 (22.4%) | 8 (7.8%) | 5 (7.7%) |  |
| Tumour size |  |  |  |  |
| T0 | 1 (1.0%) | 4 (3.9%) | 1 (1.5%) | <0.001 |
| T1 | 55 (56.1%) | 57 (55.9%) | 24 (36.9%) |  |
| T2 | 21 (21.4%) | 35 (34.3%) | 25 (38.5%) |  |
| T3 | 4 (4.1%) | 2 (2.0%) | 11 (16.9%) |  |
| T4 | 2 (2.0%) | 2 (2.0%) | 1 (1.5%) |  |
| Tis | 8 (8.2%) | 2 (2.0%) | 1 (1.5%) |  |
| Missing | 7 (7.1%) | 0 (0.0%) | 2 (3.1%) |  |
| Node Infiltration |  |  |  |  |
| N0 | 52 (53.1%) | 58 (56.9%) | 20 (30.8%) | <0.001 |
| N1 | 24 (24.5%) | 32 (31.4%) | 22 (33.8%) |  |
| N2 | 7 (7.1%) | 6 (5.9%) | 17 (26.2%) |  |
| N3 | 3 (3.1%) | 4 (3.9%) | 4 (6.2%) |  |
| Missing | 12 (12.2%) | 2 (2.0%) | 2 (3.1%) |  |
| Metastasis |  |  |  |  |
| M0 | 83 (84.7%) | 102 (100.0%) | 65 (100.0%) | <0.001 |
| M1 | 1 (1.0%) | 0 (0.0%) | 0 (0.0%) |  |
| Missing | 14 (14.3%) | 0 (0.0%) | 0 (0.0%) |  |
| Tumour stage |  |  |  |  |
| I | 43 (43.9%) | 41 (40.2%) | 12 (18.5%) | <0.001 |
| II | 30 (30.6%) | 46 (45.1%) | 23 (35.4%) |  |
| III | 11 (11.2%) | 10 (9.8%) | 22 (33.8%) |  |
| IV | 1 (1.0%) | 0 (0.0%) | 0 (0.0%) |  |
| Missing | 13 (13.3%) | 5 (4.9%) | 8 (12.3%) |  |
| Oestrogen receptor |  |  |  |  |
| Negative | 10 (10.2%) | 17 (16.7%) | 21 (32.3%) | 0.002 |
| Positive | 84 (85.7%) | 85 (83.3%) | 43 (66.2%) |  |
| Missing | 4 (4.1%) | 0 (0.0%) | 1 (1.5%) |  |
| Progesterone receptor | |  |  |  |
| Negative | 21 (21.4%) | 25 (24.5%) | 27 (41.5%) | 0.011 |
| Positive | 73 (74.5%) | 77 (75.5%) | 37 (56.9%) |  |
| Missing | 4 (4.1%) | 0 (0.0%) | 1 (1.5%) |  |
| Her2 |  |  |  |  |
| Negative | 70 (71.4%) | 82 (80.4%) | 48 (73.8%) | 0.006 |
| Positive | 16 (16.3%) | 20 (19.6%) | 14 (21.5%) |  |
| Missing | 12 (12.2%) | 0 (0.0%) | 3 (4.6%) |  |
| Grade |  |  |  |  |
| Well differentiated | 25 (25.5%) | 19 (18.6%) | 4 (6.2%) | 0.004 |
| Moderately differentiated | 27 (27.6%) | 17 (16.7%) | 13 (20.0%) |  |
| Poorly differentiated | 17 (17.3%) | 21 (20.6%) | 22 (33.8%) |  |
| Missing | 29 (29.6%) | 45 (44.1%) | 26 (40.0%) |  |

**Additional file Table 9**. Characteristics of the breast cancer patients and population-based controls

|  | Controls | Breast cancer | P-value | Controls | Cases detected via screening | Disease-free cases in the follow up | Cases with active disease in the follow-up | P-value |  |
| --- | --- | --- | --- | --- | --- | --- | --- | --- | --- |
| N | 131 (32.8%) | 269 (67.2%) |  | 131 (32.8%) | 102 (25.5%) | 102 (25.5%) | 65 (16.2%) |  |  |
| Age mean (SD) | 57.8 ± 12.7 | 55.8 ± 13.2 | 0.148 | 57.8 ± 12.7 | 57.5 ± 11.7 | 54.5 ± 13.8 | 55.2 ± 14.4 | 0.174 |  |
| Education |  |  |  |  |  |  |  |  |  |
| Less than primary school | 22 (16.8%) | 35 (13.0%) | 0.103 | 22 (16.8%) | 19 (18.6%) | 10 (9.8%) | 6 (9.2%) | 0.282 |  |
| Primary school | 52 (39.7%) | 85 (31.6%) |  | 52 (39.7%) | 30 (29.4%) | 34 (33.3%) | 21 (32.3%) |  |  |
| Secondary school | 33 (25.2%) | 99 (36.8%) |  | 33 (25.2%) | 37 (36.3%) | 38 (37.3%) | 24 (36.9%) |  |  |
| University | 24 (18.3%) | 50 (18.6%) |  | 24 (18.3%) | 16 (15.7%) | 20 (19.6%) | 14 (21.5%) |  |  |
| Body mass index (kg/m2) |  |  |  |  |  |  |  |  |  |
| < 18.5 | 2 (1.5%) | 2 (0.7%) | 0.861 | 2 (1.5%) | 1 (1.0%) | 0 (0.0%) | 1 (1.5%) | 0.397 |  |
| 18.5–24.9 | 64 (48.9%) | 132 (49.1%) |  | 64 (48.9%) | 44 (43.1%) | 60 (58.8%) | 28 (43.1%) |  |  |
| 25–29.9 (overweight) | 44 (33.6%) | 87 (32.3%) |  | 44 (33.6%) | 37 (36.3%) | 24 (23.5%) | 26 (40.0%) |  |  |
| ≥30 (obesity) | 21 (16.0%) | 48 (17.8%) |  | 21 (16.0%) | 20 (19.6%) | 18 (17.6%) | 10 (15.4%) |  |  |
| Menopausal status |  |  |  |  |  |  |  |  |  |
| Postmenopausal | 93 (71.0%) | 154 (57.2%) | 0.008 | 93 (71.0%) | 71 (69.6%) | 48 (47.1%) | 35 (53.8%) | <0.001 |  |
| Premenopausal | 38 (29.0%) | 115 (42.8%) |  | 38 (29.0%) | 31 (30.4%) | 54 (52.9%) | 30 (46.2%) |  |  |
| Smoking |  |  |  |  |  |  |  |  |  |
| No smoker | 74 (56.5%) | 159 (59.1%) | 0.561 | 74 (56.5%) | 61 (59.8%) | 55 (53.9%) | 43 (66.2%) | 0.312 |  |
| Former smoker | 32 (24.4%) | 70 (26.0%) |  | 32 (24.4%) | 28 (27.5%) | 32 (31.4%) | 10 (15.4%) |  |  |
| Current smoker | 25 (19.1%) | 40 (14.9%) |  | 25 (19.1%) | 13 (12.7%) | 15 (14.7%) | 12 (18.5%) |  |  |
| Alcohol consumption at recruitment (g/day) | 4.4 ± 7.6 | 5.5 ± 11.0 | 0.351 | 4.4 ± 7.6 | 4.8 ± 7.5 | 5.8 ± 12.9 | 6.0 ± 12.4 | 0.685 |  |
|  |  |  |  |  |  |  |  |  |  |
